# DC-CIK biotherapy for a recurrent benign lymphoepithelial lesion of the salivary gland: A case report and literature review

**DOI:** 10.3892/etm.2014.1937

**Published:** 2014-08-27

**Authors:** DEJUN XING, YUEMING ZHAO

**Affiliations:** Department of Medical Oncology, Jilin Cancer Hospital, Changchun, Jilin 130012, P.R. China

**Keywords:** benign lymphoepithelial lesion, dendritic cell, cytokine-induced killer cell, adoptive cellular immunotherapy

## Abstract

A benign lymphoepithelial lesion (BLEL) is an idiopathic inflammation of the parotid gland, characterized by hyperplasia, lymphocyte infiltration and the formation of epimyoepithelial islands, as well as atrophy of the gland parenchyma. Common treatment methods include immunosuppression and glucocorticoid therapies, in addition to surgical dissections. Cytokine-induced killer (CIK) cells sensitized to specific antigens by dendritic cells (DCs) are used in DC-CIK biotherapy. The present study reports the case of a 22-year-old female suffering from a postoperative recurrent BLEL on the left parotid gland, which was gradually increasing. Following initial unsuccessful conservative treatment attempts, a 10-day course of DC-CIK therapy was initiated, after which the lesion in the gland area was reduced in size and local infection and skin ulcerations were improved. DC-CIK biotherapy was continued for three months (four sessions of 10-day treatments with a 10-day break in between) until the lesion disappeared and the skin ulceration was healed. Computerized tomography scans of the parotid gland revealed complete remission of the primary lesion and recovery of the bone destruction. The patient was discharged and remained stable with no sign of recurrence during a 10-month follow-up period. In the present case report, a successful DC-CIK adoptive cellular immunotherapy treatment for a BLEL was described for the first time.

## Introduction

Benign lymphoepithelial lesions (BLELs) show three histological characteristics: Hyperplasia and infiltration of lymphocytes into the gland, atrophy of the gland parenchyma and hyperplasia and infiltration of epimyoepithelial islands in the gland duct. In 1952, Godwin denominated the condition as ‘benign lymphoepithelial lesion’ (1), which was newly classified as a benign salivary gland tumor in 1991 (2). BLELs were not considered to be tumors, but rather a type of reactive hyperplasia, characterized by a benign, long progression, which was not life-threatening and was rarely malignant in patients. BLELs appear mainly in unilateral or bilateral parotid tissues and the submandibular gland; however, cases of lymphoepithelial lesions of the salivary gland are rare, comprising <3% of benign salivary gland tumors. At present, no standard therapy regimen is available for BLELs; however, glucocorticoid therapy, such as the administration of cyclophosphamide with prednisone, as an alternative to surgery has been reported (3,4). A recent study demonstrated that BLELs are sensitive to radiotherapy, which may be beneficial in recurrent lesions (5). Although surgery and radiation are the main treatment methods (6–9), BLELs of the salivary gland have been rarely studied and no common therapy criteria or adoptive cellular immunotherapy have been reported.

## Case report

A 22-year-old female was admitted to the Jilin Cancer Hospital (Changchun, China) in May 2013. In January 2009, a 2.0×3.0 cm painless mass was surgically removed from the left parotid gland of the patient. Postoperative pathology characterized the mass as a lymphocytic hyperplasia; however, parotid duct cells were also detected within the lymphocytes. Morphological results and immunohistochemical staining supported the diagnosis of a BLEL.

### BLEL recurrence and long term development

**Figure 1:**
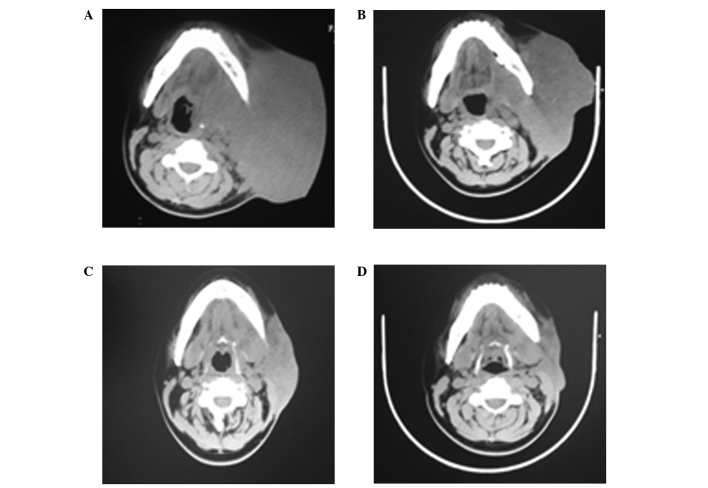
Computerized tomography images of the benign lymphoepithelial lesion in the left parotid gland (A) prior to treatment on May 8 2012, during the treatment on (B) Jun 12 2012 and (C) July 16 2012, and (D) after treatment on Sep 5 2012.

After six months, the left parotid gland became locally swollen again and intermittent management with traditional Chinese medicine was unable to achieve remission. In October 2010, the size of the mass reached 5.0×6.0 cm. A computerized tomography (CT) scan of the parotid gland indicated a soft tissue mass in the left neck area without uniform density or a clear border with the left parotid gland, and evident infiltration in the surrounding tissue. The mass was considered to be a recurrence of the BLEL in the left parotid gland; however, the patient did not agree to further surgical therapy. The mass in the left parotid gland area became gradually enlarged, and was more evident when the patient became ill with a cold; however, the mass was slightly reduced following anti-infective treatment (mainly with cephalosporins). The patient also received traditional Chinese medicine for six months and local acupuncture therapy for 40 days. However, the mass in the left parotid gland area was continually increasing in size and reached a diameter of 15 cm. On April 27 2013, the patient developed a fever of up to 40°C, accompanied with shivering, weakness and a loss of appetite, which was more severe in the afternoon and at night. Remission was observed following self-medication with oral ibuprofen; however, the temperature of the patient increased after several hours and a light yellow, clear liquid was discharged from the ulceration on the skin surface of the left parotid gland mass. On May 4 2013, the patient visited the Jilin Cancer Hospital, where the local ulceration and infection of the left parotid gland mass was managed with anti-infective treatment for three days; however, the high temperature of the patient did not decrease and the mass did not reduce in size. The patient was admitted to the hospital for further therapy on May 8 2013, and physical examination identified a large mass of 17×14 cm on the left side of the patient’s face, a red and swollen skin surface and multiple ruptures in the middle of the mass with a light yellow liquid discharge. The peripheral skin of the mass was tenacious and swollen, and the skin temperature was high. A blood routine examination and liver and renal functions were found to be normal. Color ultrasound revealed a visible 18.2×8.5-cm low-echo area with strong-echo stripes under the skin on the left side of the patient’s face, without the appearance of a clear margin. Color Doppler flow imaging revealed an abundant blood flow in the mass and the absence of normal parotid gland tissue, indicating a solid space-occupying lesion on the left parotid gland. A CT scan of the left parotid gland revealed an abnormal mass in a low-density image and an undefined margin of the lesion, with the superior border extending towards the orbit, the inferior border at the submaxillary level, the inner border at the left parapharyngeal space and the outside border at the skin of the left-side of the face. The mass reached 13.4×9.7 cm in an axial view, showing uniform density and a CT value of 47 HU (Fig. 1A), with the left parotid gland unable to be distinguished. The lesion was wrapped around the left sphenoid bone and mandible. In addition, morphological changes were observed, indicating recurrence of the BLEL in the left parotid gland with bone resorption of the sphenoid bone and mandible. A bacterial culture of the mass discharge indicated a *Staphylococcus aureus* infection, which was sensitive to levofloxacin. The body temperature of the patient returned to normal levels following the administration of levofloxacin for one week. A biopsy was not performed due to the weakness, high fever, local swelling and infection of the patient, as well as the abundant blood flow detected in the mass with color ultrasound. No consensus was reached with regard to further surgical intervention. Drugs inhibiting vascular endothelial growth (Endostar and Shenyi capsules) were administered for five days, but were discontinued by the patient due to local pain and swelling. Chemoradiotherapy was subsequently considered; however, due to the young age of the patient and the examination results not indicating malignance, the patient did not agree to the therapy.

### Dendritic cell-cytokine-induced killer cell (DC-CIK) biotherapy

Following failed conservative treatment, DC-CIK therapy was initiated based on the immune index result of the patient, which found the percentage of inhibitory T cells to be 32.2% (CD8^+^CD28^−^; normal range, 9.8–21.8%) and the CD4^+^/CD8^+^ ratio to be 0.42 (Table I), indicating low immunity. The patient signed informed consent for the treatment protocol, which was approved for tumor patients by the Health Department of Jilin Province (Changchun, China) and the Ethics Committee of the Jilin Cancer Hospital.

### Source of cells

Antigen-presenting DCs and CIK cells were produced using heat shock protein-70, extracted from a head and neck squamous carcinoma cell strain supplied by the Jilin Cancer Hospital, and cocultured with cord blood DCs.

### DC-CIK protocol

The duration of the first treatment session was 10 days, after which the treatment was discontinued for 10 days. Following the initial treatment course, the lesion in the left parotid gland area was reduced, while the local infection and skin ulceration were soothed. Biotherapy was continued for three months (four sessions of treatment), after which the left parotid gland lesion disappeared and the skin ulceration was healed.

### Treatment outcome

Parotid gland CT scans (Fig. 1B–D) indicated that the primary lesion of the left parotid gland area disappeared gradually and the bone destruction was completely recovered. The patient was discharged from hospital and the condition remained stable without signs of recurrence during a follow-up period of 10 months; however, the patient remains under close observation.

## Discussion

BLELs, also known as Mikulicz disease (MD), were first reported by Mikulicz in 1888 and are characterized by chronic, painless and symmetric swelling of tear and salivary glands (10–12). When a BLEL is combined with certain systematic diseases, including sarcoidosis leukemia, viral infections and macroglobulinemia, the condition is also known as Mikulicz syndrome. In 1933, the Swedish optician, Sjögren, identified a chronic systematic disease, known as Sjögren’s syndrome (SS), which primarily involved the salivary and tear glands (13). SS manifests as a dry mouth and xerotic keratitis, and occasionally occurs alongside other chronic connective tissue diseases. Due to the similar histological and pathological findings in MD and SS patients, Morgan and Raven proposed that MD is a subdisease of SS (14). Mihas *et al* hypothesized that MD, BLEL and SS may be conditions of the same disease process, with malignant lymphoma being a common complication (15). Due to the symptom similarities, the diagnosis of a BLEL is mainly based on clinical observations. An homogeneous, painless mass with a clear boundary at the tear gland, the absence of bone destruction and accompanied by a swollen salivary gland and dry mouth, can be diagnosed as a BLEL. However, prior to BLEL diagnosis, dry eyes, systematic connective tissue diseases and lymphoma and inflammatory pseudotumors should be excluded. In addition, diffuse hyperplasia and lymphocyte infiltration of the gland, atrophy of the gland parenchyma and the formation of epimyoepithelial islands in the gland duct should be pathologically confirmed (4).

The etiology of BLELs remains unclear. Tsubota *et al* revealed that although the pathologies of MD and SS are similar, the lacrimal gland acinar cells in SS patients were apoptotic with high protein expression levels of Fas and FasL, in contrast to the acinar cells of MD patients (16). In addition, Yamamoto *et al* observed that the serum concentration of the IgG4 antibody in BLEL patients was much higher when compared with the SS patients, and hypothesized that BLELs are a systematic disease correlating with IgG4 (17). Gupta *et al* revealed that the BLEL epimyoepithelial islands were predominantly surrounded by B lymphocytes and partially by T lymphocytes, indicating that humoral immunity was involved in BLELs (18). Initial T cell blood analysis of the patient in the present study revealed a high percentage of total T lymphocytes (CD3^+^), suppressor T cells (CD3^+^CD8^+^) and inhibitory T cells (CD3^+^CD28^−^), while a low CD4^+^/CD8^+^ ratio and number of regulatory T cells were observed. CD3^+^CD8^+^ cells have been identified as malignant in 85% of granular lymphocyte leukemias (19), and previous studies have revealed that large granular lymphocyte leukemias are strongly associated with SS, in addition to other autoimmune diseases (20–22). A low CD4^+^/CD8^+^ ratio, also observed in HIV-infected patients, is generally a sign of a weak immune system. Thus, in combination with the abnormally high percentages of inhibitory and suppressor T cells, the patient in the present study was considered to have a weakened immune system. In order to strengthen the patient’s immunity, DC-CIK therapy was attempted, which had been previously used in the treatment of non-small cell lung cancer (23–25). As a result, the percentages of suppressor, inhibitory and regulatory T cells returned to within the normal ranges and the CD4^+^/CD8^+^ ratio increased. However, the total T lymphocyte percentage decreased to below the normal range. Although the exact hematological mechanisms remain unclear and further analysis is required, considering the outcome of this first trial, DC-CIK therapy may be a promising approach for the treatment of BLELs.

## Figures and Tables

**Table I:** Changes in the T cell subpopulations during DC-CIK adoptive cellular immunotherapy treatment.

Analysis date	Total T lymphocytes^a^ (CD3^+^), %	Suppressor T cells^b^ (CD3^+^CD8^+^), %	Inhibitory T cells^c^ (CD3^+^CD28^−^), %	Regulatory T cells^d^ (CD4^+^CD25^+^), %	Immune status^e^ (CD4^+^/CD8^+^ ratio)
Jun 8 2012	79.2	59.3	32.2	0.7	0.42
Jun 28 2012	55.7	38.6	28.8	1.2	0.47
Jul 17 2012	35.7	18.1	14.8	1.0	0.87
Sep 5 2012	54.3	30.1	20.8	1.7	0.68

Normal ranges:

a 62.5–79.5%;

b 19.9–34.5%;

c 9.8–21.8%;

d 1.36–5.48%;

e 1.20–2.76%.

DC-CIK, dendritic cell-cytokine-induced killer cell.
